# Transactions of the Chicago Society of Physicians and Surgeons

**Published:** 1875-01-15

**Authors:** R. E. Starkweather


### Society Reports.

### TRANSACTIONS OF THE CHICAGO SOCIETY OF PHYSICIANS AND SURGEONS.

### REGULAR MEETING, DECEMBER 28, 1874.

*Reported by R. E. Starkweather, M.D.*

THE society met as usual in the Grand Pacific Hotel, the President in the chair. The minutes of the preceding meeting were read and approved.

Dr. W. C. Lyman proposed the name of Dr. O. C. De Wolff, (Howard Medical School) for membership.

Dr. J. H. Etheridge then reported a case of dysmenorrhœa, relieved by topical applications of the Faradic current from a Kidder's battery.

Dr. F. H. Davis reported a case of relapsing fever. The patient, with the exception of some gastric disturbance, seemed to have fairly entered upon convalescence when a relapse occurred. Quinia had been freely administered. The speaker inquired whether other cases had occurred in the practice of those present, and also whether there were cases of the relapsing fever known in Europe.

Dr. Bartlett reported a similar case in which the patient relapsed every Thursday for ten weeks, in spite of large doses of quinia. When blue mass was combined with the latter, some improvement occurred, but large doses were necessary. The patient was finally removed from his residence, when it was discovered that there was an overflow from the main sewer of the house. After the removal, recovery was complete and rapid. He had no doubt, that relapsing fever occurred in this country.

Dr. Davis explained that his patient lived in an upper story of the house, and that the wife of the latter was threatened with a similar disease.

Dr. Henrotin reported a case of emansio mensium from vaginal contraction, relieved by surgical interference. The amount of grumous blood discharged was very unusual.

Dr. A. R. Jackson reported several cases of subinvolation of the uterus, treated in the Woman's Hospital of Illinois, by the application of fuming nitric acid. He dwelt upon the normal and abnormal process, when subinvolation existed. Comparing the relative dimensions of the organ in its contracted and enlarged conditions, he explained the method employed by him in making these applications, which were painless when no sound tissue was invaded. The effect produced, was the stimulation of the womb to a point where contraction was induced. Ergot had been coincidentally administered by the mouth. Prompt and permanent cures were effected.

The Secretary made some remarks upon cases of contagious impetigo recently treated by him. He alluded to the fact, that no parasites had been found to occasion the disease, either in New York or Vienna; and announced that Dr. Tilbury Fox had recently limited to this disease, the once generic term, "Impetigo." After a brief description of the disease as it occurred in adults and children, he compared the observations of Dr. R. W. Taylor, of New York, with those of other dermatologists.

Dr. Jackson reported a case of sigmoid-shaped enlargement of the sterno-cleido-mastoid in a child.

On motion of Dr. M. W. Wood, U. S. A., the following preamble and resolutions were adopted:

"WHEREAS, Dr. Jno. Bartlett did, on the 10th of November, 1873, read to this society, a paper giving a 'description of a marsh plant from the Mississippi River ague bottoms, with a consideration of its genetic relations to malarial diseases;' and

"*Whereas,* This paper has since been published as one of the productions of this society; and

"*Whereas,* The theories advanced in this paper, have not since received, so far as known to us, either confirmation or refutation; and

"*Whereas,* It is desirable in the interests of medical science, that a matter of such importance be given a careful investigation; therefore, be it

"*Resolved,* That a committee of at least five members of this society be chosen—of whom two, at least, shall be microscopists—with instructions to wait upon Dr. Bartlett, and carefully investigate such evidence as he may have to offer, bearing upon the theories advanced by him; and to report in writing, at their earliest convenience, the result of this investigation."

The following committee was then chosen to act in accordance with the terms of the resolution:

Drs. H. A. Johnson, J. W. Freer, Walter Hay, Lester Curtis, and J. H. Etheridge.

The society then adjourned.

---

THE NEW YORK MEDICAL RECORD. — After January 1st, *The Medical (N. Y.) Record* will consist of sixteen pages, weekly, of text, and will form an annual volume of 832 royal octavo pages; being nearly one-third larger than before. The subscription price will be raised to $5 a year, and the publishers will prepay the postage to all subscribers. — *American Medical Weekly.*

MERCURIAL OINTMENT IN BOILS AND CARBUNCLES.—Dr. T. Roth lauds, in the *Deutsche Klinik,* the local application of gray ointment in boils and carbuncles, especially the early stages. He anoints the affected part with the ointment four times daily, and thereby reduces the inflammation and "backens" the boil most satisfactorily.—*Phil. Med. and Surg. Rep.*

